# Effect of Preoperative Clear Liquid Consumption on Postoperative Recovery in Pediatric Patients Undergoing Minimally Invasive Repair of Pectus Excavatum: A Prospective Randomized Controlled Study

**DOI:** 10.3390/jcm13123593

**Published:** 2024-06-19

**Authors:** Jaewon Huh, Jung-Min Koo, Minju Kim, Hoon Choi, Hyung-Joo Park, Gong-Min Rim, Wonjung Hwang

**Affiliations:** 1 Department of Anesthesiology and Pain Medicine, Seoul St. Mary’s Hospital, College of Medicine, The Catholic University of Korea, Seoul 06591, Republic of Korea; ether@catholic.ac.kr (J.H.); miniyaa623@gmail.com (J.-M.K.); minju1025@daum.net (M.K.); hoonie83@catholic.ac.kr (H.C.); 2 Department of Thoracic and Cardiovascular Surgery, Nanoori Hospitals, Seoul 06048, Republic of Korea; hyjpark@catholic.ac.kr; 3 Department of Thoracic and Cardiovascular Surgery, St. Vincent’s Hospital, College of Medicine, The Catholic University of Korea, Suwon 16247, Republic of Korea; yim8585@hanmail.net

**Keywords:** emergence delirium, pectus excavatum, pediatric surgery, preoperative fasting

## Abstract

**Background/Objectives**: Preoperative fasting guidelines traditionally aim to reduce pulmonary aspiration risk. However, concerns over the adverse effects of prolonged fasting have led to exploring alternatives. This study aimed to investigate the impact of preoperative clear liquid intake on postoperative outcomes in children undergoing minimally invasive repair of pectus excavatum (MIRPE). **Methods**: A prospective randomized controlled study was conducted on children aged 3–6 years scheduled for elective MIRPE. Patients were randomized into either a routine overnight fasting group (NPO) or a clear liquid group. The incidence and severity of emergence delirium (ED) were assessed using Pediatric Anesthesia Emergence Delirium (PAED) and Watcha scales at recovery room. Postoperative pain scores and opioid requirements were evaluated at intervals of 1–6 h, 6–12 h, and 12–24 h after surgery. **Results**: Fasting time was 178.6 ± 149.5 min and 608.9 ± 148.4 min in the clear liquid group compared and NPO group, respectively. The incidence of ED, measured by PAED and Watcha scales, was lower in the clear liquid group (PAED score ≥ 12: 55.6% vs. 85.2%, *p* = 0.037; Watcha score ≥ 3: 51.9% vs. 85.2%, *p* = 0.019). The highest PAED score recorded in the recovery room was significantly lower in the clear liquid group (11.4 ± 2.8 vs. 14.6 ± 2.8, *p* < 0.001). Clear liquid group showed significantly lower pain scores at 1–6, 6–12, and 12–24 h postoperatively. Additionally, clear liquid group had lower opioid requirement at 1–6 and 6–12 h postoperatively. **Conclusions**: Preoperative clear liquid consumption was associated with a lower incidence of ED in pediatric patients undergoing MIRPE.

## 1. Introduction

Pectus excavatum is the most prevalent congenital chest wall deformity and predominantly affects males. It requires surgical intervention to alleviate cardiopulmonary compromise and cosmetic concerns. Typically associated with adolescents around the age of 15 [1], minimally invasive repair of pectus excavatum (MIRPE) has become increasingly feasible in children aged over 3 years [2,3]. MIRPE involves minimal incisions without rib cartilage resection, unlike traditional procedures, and carries minimal risk for complications such as pneumothorax, bar dislocation, and the need for reoperation. Early surgical correction offers the added benefit of averting chest wall asymmetry, growth impediments, and psychological distress.

Preoperative fasting is essential to prevent pulmonary aspiration of gastric contents during general anesthesia. However, the conventional midnight nil per os (NPO) guideline may disrupt homeostasis, leading to fluid imbalance and increased insulin resistance. There have been concerns about prolonged fasting in pediatric patients, who have a lower risk of pulmonary aspiration compared with adults. Currently, the “6-4-2 rule” is used to guide fasting in pediatric patients undergoing elective surgery or procedural sedation: 6 h for a light meal, non-human milk, and infant formula; 4 h for breast milk; and 2 h for clear liquids [4]. Shorter fasting periods can reduce dehydration and discomfort, potentially improving preoperative conditions without increasing the risk of aspiration. Recent discussions and studies suggest even shorter fasting times as little as 1 h for clear liquids [5,6]. However, these suggestions are still under investigation, and the current evidence supporting such changes is not yet strong enough to alter standard practice widely. Studies have shown that consuming a small amount of clear fluid within 2 h before surgery reduces gastric volume without increasing aspiration risk in pediatric patients. It also facilitates recovery by alleviating postoperative nausea and vomiting (PONV), as well as pain [7,8]. 

Emergence delirium (ED) is another factor affecting postoperative recovery in children [9]. ED is characterized by impaired awareness, disorientation, and perceptual alterations, leading to stimulus hypersensitivity and hyperactive motor behavior after anesthesia. Although it is brief, ED poses significant risks, including self-harm, drain removal, wound complications, pain exacerbation, and increased need for intensive care. Fasting status, patient age, surgical duration, and anesthesia type have been implicated in the onset of ED among children [10,11]. However, current research regarding the relationship between fasting status and ED is mainly focused on minor surgeries or procedural sedation, with limited evidence in major surgery. We hypothesized that preoperative intake of clear liquids by pediatric patients undergoing MIRPE would decrease the incidence of ED and improve the quality of postoperative recovery. Therefore, the present study investigated whether preoperative ingestion of clear liquid could enhance postoperative recovery in pediatric patients undergoing MIRPE.

## 2. Materials and Methods

### 2.1. Study Design and Participants

This prospective randomized controlled study was conducted in a tertiary university hospital and adhered to the Declaration of Helsinki. The study protocol was approved by the Institutional Review Board on 28 May 2019 (KC19ENSI0335) and registered with the Clinical Research Information Service on 17 July 2019 (KCT0007350).

Children aged 3–6 years scheduled for elective MIRPE were enrolled in the study from July 2019 to December 2022. The exclusion criteria were American Society of Anesthesiology class III or higher, psychiatric or developmental disorders, chronic analgesic use, previous surgeries, and patient or caregiver refusal to participate. Patients with a history of esophageal or gastro-intestinal enteropathies, related surgeries, achalasia, esophageal stricture, or pediatric diabetes mellitus with gastroparesis were also excluded because these factors are associated with delayed gastric emptying time. Written informed consent was obtained on the day before surgery from each patient’s parents.

### 2.2. Randomization and Blinding

Based on random computer-generated numbers, children were randomly assigned to either the routine overnight fasting group (NPO group; odd numbers) or the clear liquid group (even numbers). Patients in the NPO group underwent conventional overnight fasting, whereas clear liquid group patients consumed 3 mL/kg of water 2 h before surgery. Patients and caregivers could not be blinded; however, the attending surgeons, anesthesiologists, and healthcare providers responsible for measuring postoperative outcomes were blinded to group allocation. 

### 2.3. General Anesthesia and Surgical Procedure

Patients were sedated in the waiting room using 1–2 mg/kg of intravenous ketamine (Ketamine HCl^®^, Huons, Seoul, Republic of Korea) and 0.005 mg/kg of glycopyrrolate (Morbinul^®^, Myungmoon Pharm. Co., Ltd., Seoul, Republic of Korea). Upon entry into the operating room, standard anesthesia monitoring was initiated, including pulse oximetry, electrocardiogram, non-invasive blood pressure monitoring, and bispectral index (BIS). An induction dose (0.5–1 mcg/kg/min) of remifentanil (Ultiva^®^, GlaxoSmithKline, Brentford, UK) and 0.6 mg/kg of rocuronium (Esmeron^®^, MSD Korea, Seoul, Republic of Korea) was administered for endotracheal intubation. To prevent PONV, 0.5 mg/kg of dexamethasone and 0.02 mg/kg of palonosetron were injected at the start and end of anesthesia, respectively. After endotracheal intubation, anesthesia depth was maintained using 1.5–2% sevoflurane (Sevorane^®^, AbbVie Korea, Seoul, Republic of Korea) at a BIS of 40–60. The rate of remifentanil infusion was adjusted to maintain systolic blood pressure and heart rate within ±20% of preoperative values. Mechanical ventilation was performed with a tidal volume of 4–6 mL/kg to maintain an end-tidal CO_2_ of 35–40 mmHg. At the time of skin suturing, remifentanil infusion was discontinued, the sevoflurane dose was reduced by half, and muscle relaxant reversal was achieved using 0.15–0.2 mg/kg of pyridostigmine (Pyrinol^®^, Kukje Pharma, Seoul, Republic of Korea) and 0.02 mg/kg of glycopyrrolate. Endotracheal extubation was then performed, and children were transferred to the recovery room for postoperative care.

The surgical procedures in this study were conducted exclusively by a single surgeon who tailored the technique to each patient’s unique anatomical features and disease severity. Prior to surgery, a thoracic surgeon reviewed computed tomography (CT) scans of the patient to assess the severity of chest wall depression using the Haller and symmetry indexes. This allowed the selection of pectus bars with appropriate size and shape, customized to each patient’s chest morphology. 

Patients were positioned supine, and general anesthesia was administered. Specialized arm slings were utilized to suspend both arms overhead, ensuring an optimal surgical field. The insertion point for the pectus bar was meticulously identified based on the depressed area and bilateral hinge points on the patient’s chest. Pectus bars (Pectus Bar^®^, Biomet Microfixation, Jacksonville, FL, USA) were shaped directly at the operating table, considering the patient’s chest wall morphology. The size of the pectus bar was determined using precise measurements, with nine- and ten-inch bars used depending on individual needs. In cases of extensive chest wall depression, multiple bars were strategically employed. Small midaxillary skin incisions were made on both sides of the chest to introduce the pectus bar. Utilizing pectoscopic visualization and a 20 Fr chest tube as a guide, the bar was carefully passed below the sternum and guided across the mediastinum. The bar was flipped 180° using a specialized flipper to elevate the depressed chest wall and restore anatomical alignment. Stability was ensured by securing the bar in place with claw fixators or bridge plates. Immediate anterior–posterior chest radiography in the operating room verified the accurate positioning of the bars and detected potential complications like pneumothorax. Drains were not placed unless major bleeding occurred, contributing to patient comfort and postoperative recovery.

### 2.4. Multimodal Analgesia Management

An intraoperative multimodal analgesic approach was implemented in accordance with institutional protocols [3]. At the conclusion of surgery, a thoracic continuous wound infiltration system (CWIS; On-Q^®^ Pain relief system, Halyard, Alpharetta, GA, USA) was placed subcutaneously to deliver continuous local anesthetics at a rate of 4 mL/h. The composition of the infiltration system was adjusted based on the patient’s body weight, with a mixture of ropivacaine (Naropin^®^, Aspen Pharmacare, Durban, South Africa) and normal saline (0.15% ropivacaine for ≤15 kg, 0.2% for 16–20 kg, and 0.25% for 21–25 kg). Additionally, intercostal blocks with 2 mL of 0.5–0.75% ropivacaine solution were administered at the 4th to 9th intercostal levels.

Upon arrival in the recovery unit, children received 0.5 mcg/kg of fentanyl (Fentanyl^®^, Hana Pharm, Seoul, Republic of Korea) if their pain scores met any of the following criteria: Face, Legs, Activity, Cry, Consolability (FLACC) score ≥ 4, Pediatric Anesthesia Emergence Delirium (PAED) scale score ≥ 12, or Watcha score ≥ 3. Additional doses of fentanyl were administered if agitation persisted. Detailed descriptions of each score are provided in the outcome measurement section.

Upon transfer to the ward, the CWIS system remained in place for 72 h to continuously deliver local anesthetics at 4 mL/h, and 100–150 mg oral ibuprofen (Brufen^®^, Kukje Pharma, Seoul, Republic of Korea) was administered three times per day. Pain assessment in the ward utilized the revised version of the faces pain scale (FPS-R) as a simplified method. Children and their parents received education about the FPS-R during the informed consent process. The FPS-R is a self-reported scale ranging from 0 to 10, with visual depictions of six faces that represent varying levels of pain on a two-point scale. The left-most face indicates no pain (score: 0), whereas the right-most face indicates severe pain (score: 10). The scale has been validated for appropriate use from the age of 3 [12]. Scores above 4 on the FPS-R prompted administration of 1 mg/kg of intravenous meperidine (Demerol^®^, Pfizer, New York, NY, USA), with additional doses of 0.4–1 mg/kg of intravenous ketorolac (Ketoracin^®^, Hana Pharm, Seoul, Republic of Korea) if pain persisted. 

### 2.5. Outcome Measurement

The primary outcome of the study was the proportion of patients exhibiting ED within 1 h in the recovery room. This was assessed using the PAED and Watcha scores, measured at 5, 10, 15, 30, 45, and 60 min postoperatively. The PAED score comprises five statements, each scored on a 5-point scale (from 0 to 4), with a maximum total score of 20. These statements assess various symptoms of delirium, including the ability to make eye contact, purposeful actions, awareness of the environment, restlessness, and consolability. The highest PAED score recorded among the six time points was used for analysis. To enhance data accuracy, the Watcha scale was also utilized. This simpler scale categorizes the patient’s arousal state from 0 (asleep) to 4 (agitated and thrashing around). The presence of ED was defined as a PAED score of ≥12 or a Watcha score of ≥3, and the number (proportion) of patients meeting this definition was recorded. The highest emergence agitation score observed during the recovery period was also documented.

Secondary outcomes were classified into four categories: preoperative anxiety levels, intraoperative variables, postoperative pain scores, and postoperative opioid requirement. 

#### 2.5.1. Preoperative Anxiety Level

Preoperative anxiety was assessed using the Modified Yale Preoperative Anxiety Scale (mYPAS). Before anesthesia initiation, the attending anesthesiologist evaluated each patient’s baseline anxiety level in the waiting room. The mYPAS comprises 18 statements categorized into four anxiety behaviors: four for activity, six for vocalization, four for emotional expressivity, and four for the state of arousal. The sum of scores from these behaviors indicates the level of anxiety, with scores ranging from 4 (lowest anxiety) to 18 (highest anxiety).

#### 2.5.2. Intraoperative Variables

Systolic blood pressure, diastolic blood pressure, heart rate, and mean blood pressure were recorded before anesthesia induction, at incision, 30 min after incision, and at the end of surgery. The intraoperative mean concentration of sevoflurane was calculated as “volume percentage of sevoflurane (vol%) × duration of inhalation (minutes)/total anesthesia duration (minutes)”. Additionally, the total infused amount of remifentanil was calculated and recorded as mcg/kg/h.

#### 2.5.3. Postoperative Pain Score

The highest FLACC score, assessing pain through five categories (face, legs, activity, cry, and consolability), was measured in the recovery room after surgery. The FLACC scale ranges from 0 to 10, with higher scores indicating greater pain. Subsequently, pain was evaluated using FPS-R when patients had been transferred to the ward. The FPS-R features six faces representing pain scores from 0 to 10 with 2-point increments. FPS-R assessments were conducted at 1–6, 6–12, and 12–24 h postoperatively. Patients reported their highest FPS-R score within each time interval.

#### 2.5.4. Postoperative Opioid Requirement

Opioid consumption in the recovery room (fentanyl) and ward (pethidine) was documented. The fentanyl dose in the recovery room was recorded in mcg/kg, whereas the cumulative opioid dosage in the ward was converted to morphine equivalent dose (mcg/kg) for standardized comparison.

### 2.6. Statistical Analysis

Considering the lack of previous research regarding ED incidence in the study population, preliminary data were gathered through a pilot study involving nine patients per group. The incidences of ED were 75% in the NPO group and 38% in the clear liquid group. To achieve a power of 80% and an error rate of 0.05 in the present study, 27 patients were required in each group. Therefore, 60 patients were recruited, considering a 10% dropout rate.

The normality of variable distribution was assessed using the Kolmogorov–Smirnov test. Statistical comparisons for quantitative variables were conducted using either Student’s *t*-test or the Mann–Whitney U test; categorical data were analyzed using the Pearson χ^2^ test or Fisher’s exact test. The threshold for statistical significance was regarded as *p* < 0.05. Data are presented as means ± standard deviations (SDs) for quantitative variables and as frequencies (percentages) for categorical variables. All analyses were performed using IBM SPSS Statistics for Windows (v. 26.0; IBM Corp., Armonk, NY, USA).

## 3. Results

**Figure 1:**
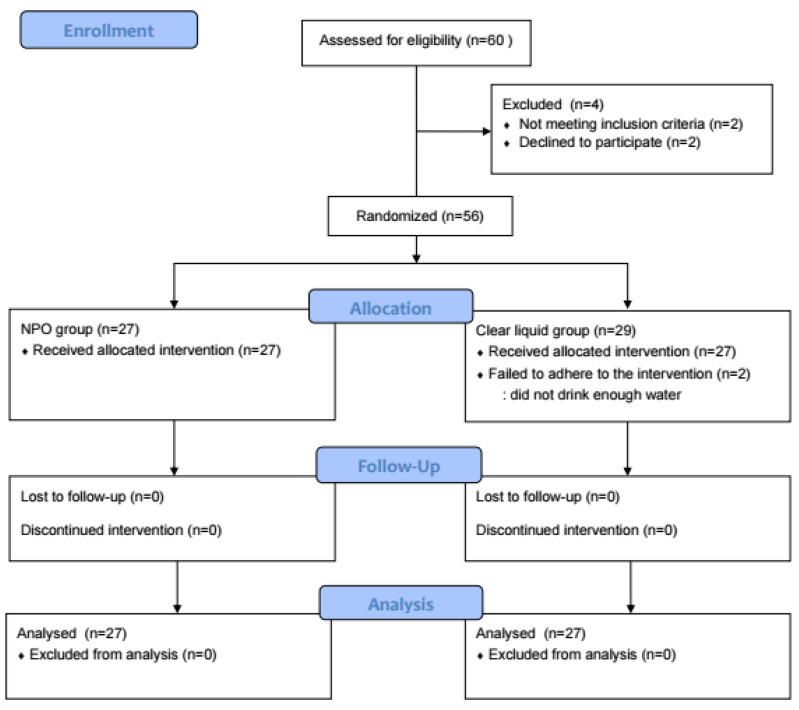
CONSORT flow diagram.

In total, 60 patients underwent randomization and 54 patients were included in the data analysis (27 per group) (Figure 1).

The mean patient age was 4.6 ± 0.9 years, and most patients (83.3%) were male. Haller index and symmetry index measurements from preoperative CT scans did not significantly differ between the two groups. The solid fasting time did not differ between the two groups. The liquid fasting time was significantly longer in the NPO group than in the clear liquid group (608.9 ± 148.4 min vs. 178.6 ± 149.5 min, *p* < 0.001). Detailed patient demographics are presented in Table 1.

**Figure 2:**
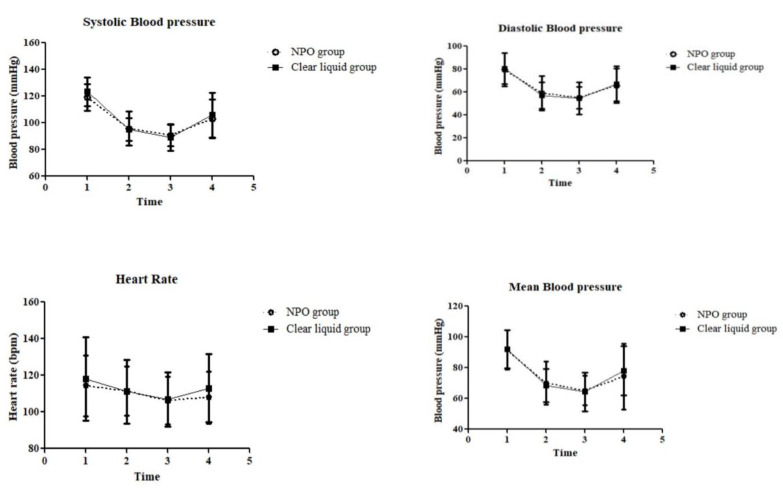
Comparison of intraoperative vital signs. Time: 1. Before anesthesia; 2. At incision; 3. 30 min after incision; 4. End of surgery.

Intraoperative vital signs did not significantly differ between the groups (Figure 2).

Intraoperative variables, including bar length, were comparable between the groups (Table 2). However, the intraoperative sevoflurane concentration and remifentanil requirement were higher in the NPO group than in the clear liquid group.

The primary outcome of the study (i.e., the incidence of ED) was higher in the NPO group than in the clear liquid group (Table 3). The proportions of patients exhibiting a PAED score of ≥12 (85.2% vs. 55.6%, *p* = 0.037) and a Watcha score of ≥3 (85.2% vs. 51.9%, *p* = 0.019) within the 60-min recovery period were greater in the NPO group. The highest recorded PAED scores in the recovery room were 14.6 ± 2.8 and 11.4 ± 2.8 for the NPO and clear liquid groups, respectively (*p* < 0.001).

There were no significant differences in peak FLACC scores, number of rescue requirement, and the amount of fentanyl administered in the recovery room between the groups. Despite similar pain scores in the recovery room, pain scores measured in the ward were consistently lower in the clear liquid group across the 1–6 h, 6–12 h, and 12–24 h periods. The cumulative opioid requirement was also lower in the clear liquid group during the 1–6 h and 1–12 h periods; however, no difference was observed at 1–24 h (Table 3).

## 4. Discussion

The present study assessed the quality of recovery in children aged 3–6 years undergoing MIRPE according to their fasting status. A higher number of patients in the NPO group exhibited ED, as determined using PAED and Watcha scores, compared with the clear liquid group. Additionally, the highest PAED score and the intraoperative requirements for sevoflurane and remifentanil were lower in the clear liquid group than in the NPO group.

Prolonged fasting in children can lead to various adverse effects, including irritability, agitation, and an increased risk of PONV, as well as more serious complications (e.g., electrolyte imbalance, hypoglycemia, and ketogenesis) [13]. Our findings were consistent with previous research indicating an elevated incidence of ED associated with extended fasting durations. In a previous study, fasting duration was associated with the incidences of ED at 15 and 20 min of recovery after ophthalmic examination [11]. Another study involving children who underwent procedural sedation for magnetic resonance imaging showed a relationship between fasting duration and ED incidence [10]. Compared with liquid fasting, solid fasting had a stronger association with increased ED rate.

The etiology of ED is multifactorial, influenced by factors such as age, type of surgery, anesthetic management, pain levels, and preoperative anxiety. However, in our study, age, surgical type, anesthetic management, postoperative FLACC scores, and preoperative anxiety levels assessed using mYPAS did not significantly differ between the two groups. Similarly, factors we previously identified as related to postoperative pain and opioid requirement, including sex, pectus bar length, Haller index, and asymmetry index [3], were comparable between the two groups.

The observed difference in ED incidence between the NPO and clear liquid groups in our study may be related to the lower concentration of sevoflurane required during surgery in the clear liquid group. Sevoflurane is considered a risk factor for ED after pediatric anesthesia [14,15,16,17]. Studies have revealed a correlation between higher sevoflurane concentrations and increased agitation incidence in children undergoing various surgical procedures. A study comparing agitation scores in children undergoing inguinal hernia repair with caudal block found that children receiving higher doses of end-tidal sevoflurane (2.5%) during face-mask sedation exhibited higher agitation rates compared with children receiving lower doses (1%) [18]. Another study demonstrated a relationship between the area under the curve of end-tidal sevoflurane concentration over time (EtSevo-time AUC) and ED. An EtSevo-time AUC of 2500–3000 in children showed a higher odds ratio (15.84) of agitation compared with lower EtSevo-time AUC values of 2000–2500 and ≤2000 (odds ratios: 3.99 and 1, respectively) [19]. We suspect that preoperative ingestion of clear liquids helped patients better cope with surgical stimuli by maintaining a euvolemic state compared to the fasting group. Preoperative ingestion of clear liquids has been shown to maintain euvolemia and prevent dehydration, ensuring better perfusion of tissues and organs [4,20]. This optimal perfusion reduces the physiological stress response to surgical stimuli by stabilizing hemodynamic parameters and reducing the release of stress hormones such as cortisol [21,22,23] Consequently, patients who are adequately hydrated preoperatively require less anesthetic and analgesic medication to achieve the same depth of anesthesia and pain control. This can be attributed to the body’s enhanced ability to manage and respond to surgical stress when in a euvolemic state. Additionally, preoperative hydration may alleviate sensations of thirst and hunger, contributing to less postoperative discomfort and a lower incidence of ED.

The incidences of ED in our study were particularly high, reaching 85.3% in the NPO group and 55.6% in the clear liquid group; both exceeded previous findings in pediatric surgery. ED rates vary among surgical procedures; ophthalmic, otolaryngologic, and strabismus surgeries exhibit higher ED rates in children aged 3–7 years [24,25]. Additionally, studies have demonstrated ED incidence rates of 61% for tonsillectomy and adenoidectomy [26], 68% for strabismus surgery [14], and 55% for pediatric nevus surgery [27]. Another study showed that up to 80% of children may experience ED after general anesthesia, and the incidence is correlated with the extent of surgery [17]. Extensive surgical procedures, including spinal, musculoskeletal, and abdominal surgeries, also reportedly increase ED risk in adult patients [28]. Our study included children undergoing major surgery, which may have contributed to the higher incidence of ED. Additionally, the predominance of male patients (83%) in our study population may have further increased the incidence of ED because male patients tend to have a 1.62-fold higher incidence compared with female patients [29,30]. Therefore, the high ED incidence in our study can be attributed to the surgeries with higher postoperative pain levels, such as pectus surgeries and the predominance of male patients.

Contrary to our initial hypothesis that administering clear liquids would decrease the incidence of postoperative side effects, the rates of PONV were similar between the two groups, remaining below 10%. This could be attributed to the implementation of multidisciplinary approaches for PONV prophylaxis at the initiation and conclusion of anesthesia, as well as the reduced opioid consumption achieved through multimodal analgesic management strategies, including intercostal blocks, CWIS, and routine non-opioid analgesic administration.

ED presents significant challenges in perioperative care due to its multifactorial nature and complex manifestations. Accurate assessment of ED is essential for effective management and improved patient outcomes. In our study, we utilized two ED evaluation scores—PAED and Watcha—with good sensitivity and specificity in diagnosing ED. Although the cut-off values for PAED have varied among studies, a PAED score of ≥12 more accurately identifies ED compared with a score of 10 [31,32]. A PAED score of ≥12 has demonstrated 100% sensitivity and 94.5% specificity in diagnosing ED [33]. Similarly, a Watcha score of ≥3 indicates the presence of ED, exhibiting a higher sensitivity and specificity compared with other ED scoring systems [34]. In our study, the number (proportion) of patients exhibiting ED was similar for both scores.

This study had several limitations. First, our study’s generalizability is limited by several factors: it was conducted at a single institution with a small sample size; all operations were performed by a single surgeon; the inclusion of children aged 3–6 years is not representative of the typical age group undergoing pectus excavatum repair; and our institution’s unique patient management protocols may differ from those in other regions or countries. Second, while we hypothesize that preoperative ingestion of clear liquids reduces stress responses and anesthetic requirements through maintaining euvolemia, we could not directly measure stress hormones or other physiological markers due to ethical constraints with our pediatric population. Third, distinguishing between ED and pain can be difficult in children due to the potential overlap and mutual influence that may affect measurement accuracy. To mitigate this effect, we implemented a multimodal analgesic technique that ensured adequate pain relief, minimizing the impact of pain on ED. Postoperative pain after MIRPE was comparable between the two groups, and trained healthcare professionals assessed the pain scores, thus enhancing measurement accuracy. Therefore, we believe that the presence of ED was measured with considerable precision. Lastly, some children in the clear liquid group were unable to strictly adhere to the water intake protocol 2 h before surgery. Difficulties arose due to the relatively large volume of fluid intake, particularly when elective procedures were scheduled for 8:00 A.M. Children were required to awaken at 6:00 A.M. to drink water; two patients were excluded from the study due to their refusal to consume the necessary amount of water. Although our findings suggested that children undergo longer preoperative fasting times than recommended by anesthesia societies, effective implementation of this finding in clinical practice requires careful consideration.

## 5. Conclusions

In conclusion, administering 3 mL/kg of water 2 h before MIRPE reduced the incidence of ED in children aged 3–6 years compared with conventional overnight fasting. This approach also mitigated the severity of ED, as reflected by anxiety scores. The reduced pain scores and opioid requirements in the clear liquid group indicated improved postoperative recovery. These findings highlight the potential benefits of implementing clear liquid protocols in pediatric surgery.

## Figures and Tables

**Table 1:** Preoperative variables.

	NPO Group (N = 27)	Clear Liquid Group (N = 27)	*p*
Age (years)	4.6 ± 0.8	4.7 ± 1.0	0.539 *
Sex (male/female)	23/4	22/5	1.000 ^†^
ASA class (1/2)	25/2	26/1	1.000 ^††^
Height (cm)	110.0 ± 6.8	110.9 ± 7.0	0.648 *
Weight (kg)	19.4 ± 3.6	19.4 ± 3.1	0.990 *
Haller index	4.5 ± 1.5	4.5 ± 1.7	0.630 *
Symmetry (Y/N)	24/3	23/4	1.000 ^††^
Fasting time (min)			
Solid	613.4 ± 146.9	573.4 ± 198.1	0.404 *
Liquid	608.9 ± 148.4	178.6 ± 149.5	<0.001 *
mYPAS	19.4 ± 3.6	19.4 ± 3.1	0.990 *

Values are expressed as numbers or mean ± standard deviation. * indicates Student’s *t*-test; ^†^ indicates Chi-square test, ^††^ indicates Fisher’s exact test. ASA: American Society of Anesthesiologists; mYPAS: modified Yale Preoperative Anxiety Scale.

**Table 2:** Intraoperative variables.

	NPO Group	Clear Liquid Group	*p*
	(N = 27)	(N = 27)	
Operation time (min)	70.7 ± 18.4	70.7 ± 18.7	0.994 *
Anesthesia time (min)	102.4 ± 14.2	106.4 ± 16.6	0.347 *
Fluid balance (mL)	92.4 ± 42.5	75.9 ± 30.6	0.108 *
Sevo (vol%)	2.3 ± 0.2	2.1 ± 0.2	0.004 *
Remifentanil (mcg/kg/h)	7.2 ± 6.9	4.0 ± 1.6	0.030 *
Pectus bar length (9/10)	22/5	19/8	0.526 ^†^

Values are expressed as numbers or mean ± standard deviation. * indicates Student’s *t*-test; ^†^ indicates Chi-square test. Fluid balance = infused volume-blood loss, Sevo: intraoperative mean concentration of sevoflurane.

**Table 3:** Postoperative variables.

	NPO Group (N = 27)	Clear Liquid Group (N = 27)	*p*
**At recovery unit**			
P-ED	23 (85.2%)	15 (55.6%)	0.037 ^†^
W-ED	23 (85.2%)	14 (51.9%)	0.019 ^†^
Highest PAED	14.6 ± 2.8	11.4 ± 2.8	<0.001 *
Highest FLACC	6.9 ± 3.3	6.1 ± 2.4	0.329 *
Rescue requirement (0/1/2)	1/22/4	2/14/11	0.055 ^†^
Rescue fentanyl (mcg/kg)	0.51 (0.48–0.57)	0.53 (0.48–0.98)	0.186 **
**At ward**			
FPS 1–6 h	6.0 (5.0–6.0)	4.0 (2.0–6.0)	0.003 **
FPS 6–12 h	5.2 ± 1.4 1.6	4.0 ± 1.9	0.011 *
FPS 12–24 h	4.8 ± 1.4	3.0 ± 1.7	<0.001 *
Cumulative opioid requirement			
1–6 h (mcg/kg)	1.0 (0.5–2.2)	0.5 (0.2–1.0)	0.039 **
1–12 h (mcg/kg)	3.2 ± 1.9	2.3 ± 1.4	0.046 *
1–24 h (mcg/kg)	5.1 ± 2.8	3.9 ± 2.5	0.117 *

Values are expressed as numbers (proportions) or mean ± standard deviation or median (interquartile range). * indicates Student’s *t*-test; ** indicates Mann–Whitney U test, ^†^ Fisher’s exact test. P-ED: the presence of ED defined as a PAED score of ≥12; W-ED: the presence of ED defined as a Watcha score ≥3, PAED: Pediatric Anesthesia Emergence Delirium scale score, FLACC: Face, Legs, Activity, Cry, Consolability score, FPS: faces pain scale.

## Data Availability

The data generated in this study can be shared after a reasonable request to the corresponding author.
